# Space Charge Modulated Electrical Breakdown

**DOI:** 10.1038/srep32588

**Published:** 2016-09-07

**Authors:** Shengtao Li, Yuanwei Zhu, Daomin Min, George Chen

**Affiliations:** 1 State Key Laboratory of Electrical Insulation and Power Equipment, Xi’an Jiaotong University, Xi’an 710049, China; 2 School of Electronics and Computer Science University of Southampton, Southampton SO17 1BJ, UK

## Abstract

Electrical breakdown is one of the most important physical phenomena in electrical and electronic engineering. Since the early 20^th^ century, many theories and models of electrical breakdown have been proposed, but the origin of one key issue, that the explanation for dc breakdown strength being twice or higher than ac breakdown strength in insulating materials, remains unclear. Here, by employing a bipolar charge transport model, we investigate the space charge dynamics in both dc and ac breakdown processes. We demonstrate the differences in charge accumulations under both dc and ac stresses and estimate the breakdown strength, which is modulated by the electric field distortion induced by space charge. It is concluded that dc breakdown initializes in the bulk whereas ac breakdown initializes in the vicinity of the sample-electrode interface. Compared with dc breakdown, the lower breakdown strength under ac stress and the decreasing breakdown strength with an increase in applied frequency, are both attributed to the electric field distortion induced by space charges located in the vicinity of the electrodes.

Electrical breakdown is an important physical phenomenon, which may occur in electrical equipment and electronic devices. Since the application of alternating current (ac) in power transmission began in the late 19^th^ century, it has been realized that the dc breakdown strength is about twice or higher than the ac breakdown strength in insulating materials^1^. Throughout the 20^th^ century, many investigations on this phenomenon were conducted^1^,^2^,^3^,^4^,^5^,^6^,^7^,^8^,^9^,^10^ and it was discovered that this phenomenon commonly exists in many insulating materials, from thin films to thick boards^3^. From the early 20^th^ century on, now-classical theories such as electrical breakdown (introduced by Hipple in 1937^11^ and Fröhlich in 1939^12^) and thermal breakdown^13^ (introduced by Wagner in 1922) have been proposed. However, it is still the lack of a widely recognized understanding, which can fully explain the origin of this phenomenon. Given overall rapid developments in electrical and electronic engineering, the incomplete understanding of the mechanism of dc and ac breakdown has restricted the development of some electrical equipment and electronic devices^4^,^5^,^6^,^9^.

In 1914, scholars first recognized the presence of charges injected from electrodes under electrical stress, i.e., space charges^14^. After the establishment of pulsed electro-acoustic (PEA) method in 1990s, space charge distribution in solid insulating materials came to be intuitively demonstrated^15^. During the same period, numerical simulations of space charge profiles were initially performed^16^,^17^ and some of the simulated results could perfectly match the PEA results in recent years^18^. As a result of the development in understanding space charges, the electric field concentration and distortion induced by space charges were valued and considered as an important factor that modifies the dielectric behaviour of insulating materials^19^,^20^. Based on present understanding, the origin of thickness-dependent dc breakdown was initially investigated through numerical simulations^21^. However, there is still a lack of experimental support. Moreover, the origin of ac breakdown in connection with the presence of space charge has not been revealed.

In this work, after considering the characteristics of charge injection, migration and diffusion, we demonstrate the charge transport behaviours during dc and ac (50–1000 Hz) breakdown of one typical material employed in electrical engineering, i.e. the oil impregnated paper in power transformers. We discuss the differences in charge accumulation and electric field distortion between dc and ac electrical stresses, and reveal the breakdown mechanism modulated by space charges.

Our experiment employed Karamay 25^#^ transformer oil impregnated Kraft insulating paper (thickness of 0.07 mm) as the oil impregnated paper sample. A brass spherical electrode (*ϕ* = 25 mm) was used in conducting the breakdown tests. (Further details are provided in the Supplementary Information).

Figure 1a shows the downward trend of experimental dc breakdown strength with increasing sample layers (i.e. thickness), from 233.4 kV/mm of a one-layer sample to 217.3 kV/mm of a four-layer sample. The downward trend of dc breakdown strength is commonly acknowledged to be a geometrical effect and it is widely observed in insulating materials^22^,^23^. In order to investigate the mechanism of dc breakdown and the origin of the geometrical effect, we analyse the space charge dynamics during dc breakdown through numerical simulations^24^. The details and the assigned values of the parameters employed in the simulation are described in the Supplementary Information.

The space charge profiles for 1–4 layer samples are similar and the two-layer sample is thus taken as an example, as shown in Fig. 1b. For a two-layer sample, the experimental dc breakdown occurs at an average value of 32.12 kV. Before breakdown, a dc voltage with a ramping rate of 1 kV/s is continuously applied, injecting homo-space charges into the sample. The charge injection follows Schottky thermionic emission, which implies that the quantity of injected charges is exponentially increasing with the ramping voltage. As a result, space charge density increases with time.

Under applied dc stress, the injected space charges induce a built-in electric field, as shown in Fig. 1c. The homo-charges suppress the electric strength in the vicinity of the sample-electrode interface, while they increase the electric strength in the middle of the bulk. This electric field distortion is not apparently formed during the early breakdown process, as the quantity of accumulated charges is relatively small. However, when the applied field is approaching the dc breakdown strength, the electric field distortion increases dramatically. As electrical breakdown occurs at the position where there is the highest electric strength, dc breakdown initializes in the middle of the bulk.

Before breakdown, the duration of the application of dc stress is lengthened with increasing sample thickness. As a result, homo-charge accumulation is strengthened, which leads to a more obvious decrease of electric strength near the sample-electrode interface, as well as an enhancement in the bulk of the sample. It implies a higher maximum built-in electric strength in the bulk, which decreases the breakdown strength. As a result, the dc breakdown strength decreases with increasing sample thickness. For thickness-dependent dc breakdown of oil impregnated paper, the simulated breakdown strengths are in accordance with the experimental results, as shown in Fig. 1a.

Since the late 20^th^ century, space charge has been regarded as an important factor leading to deterioration and electrical failure of materials subjected to high voltage dc electrical stress. However, the understanding of space charge behaviours under ac stress is comparatively limited^25^ and their influence on breakdown process is still a debated topic. One main reason is that reliable experimental detection of space charges under ac stress is much harder when compared to that under dc stress, owing to the extremely fast periodic motion of applied voltage amplitudes. Moreover, a technological blockage is encountered in detecting space charge distribution on a small scale, e.g., the resolution of the latest PEA system is reported as 2 μm^26^, while space charges under ac stress are commonly continuously distributed within 0–2 μm in the vicinity of the electrodes^27^. Therefore, the space charge distribution in short-term ac breakdown is seldom demonstrated in experiments.

Considering the constraints in experimentally measuring space charge distributions under ac stress, numerical simulations are employed in academic investigations^25^,^28^. Owning to the difficultly in maintaining the convergence and stability of a simulating program, the highest reported ac frequency in simulations of space charge profiles is 50 Hz^27^. However, higher frequency voltages are commonly applied in electronic and electrical applications and their related investigations remain blank. We develop a simulating program, which combines the third-order finite difference method, the finite element method, and the reaction-advection-reaction algorithm. This program has the advantage of obtaining stabilized numerical solutions at high frequencies and it can accurately simulate space charge dynamics under 50–1000 Hz ac stress.

Figure 2a shows the experimental breakdown strengths of one-layer oil impregnated paper under 50–1000 Hz ac stress. The ac breakdown strength decreases with increasing applied voltage frequency. Under 50 Hz ac stress, the breakdown occurs at 135 kV/mm, which is 57.7% of the dc breakdown strength (233 kV/mm) of the same sample.

In order to demonstrate the electric field distortion with the variation of applied frequency, it is assumed that no breakdown occurs in the following numerical simulation. Figure 2b shows the space charge distribution under 50–1000 Hz ac stress at 135 kV/mm (the experimental 50 Hz breakdown strength, peak-to-peak value). It can be observed that both positive and negative charges are accumulated in the vicinity of the electrodes. Compared with space charge distribution under dc stress, the charge accumulation depth is tremendously narrowed, which is within 0–2 μm in the vicinity of the electrodes. Moreover, the position of positive charges is more shallow (compared to that of negative charges), owing to a lower mobility that controls the migration of charges. Although the total amount of space charges under ac stress is relatively small, the charge densities in the vicinity of the electrodes are extremely large. The maximum density reaches the order of 10^4^ C/m^3^ at the electrodes, while no space charge is observed inside the middle of the bulk. The densities of both positive and negative charges increase with increasing applied frequency, which is considered to determine the breakdown strengths with the variation of applied frequencies.

In Fig. 2b, it is observed that space charge accumulation is more dramatic in the vicinity of the left electrode, which forms a more distorted electric field. The amplitude of ac stress varies in one cycle. Electrical breakdown occurs at the maximum electric field, which is built at the phase angle of 90°, as demonstrated in Fig. 2c. It is observed that a tremendously distorted field is built near the sample-electrode interface and is strengthened with the increasing applied frequency. The maximum distortion appears at the junction of positive and negative charges, which can be explained by Poisson’s equation^24^. For 50 Hz ac stress, when the applied electric field is 135 kV/mm, the maximum electric strength reaches 233 kV/mm in the vicinity of the left electrode. As 233 kV/mm is the assigned value of intrinsic breakdown (that is, the experimental dc breakdown strength), this leads to the breakdown of the oil impregnated paper sample under 50 Hz ac stress. For 200–1000 Hz ac stress, the maximum distorted electric strengths at 135 kV/mm are far larger than 233 kV/mm and they increase with the increasing applied frequency. Therefore, the breakdown strengths decrease with the rising applied frequencies.

The simulated breakdown strengths under 200–1000 Hz ac stress are shown in Fig. 2a, which are in accordance with the experimental results. Ac breakdown is determined by space charges in the vicinity of the sample-electrode interface. The comparatively lower breakdown strength (compared with dc breakdown strength) and the decreasing trend with increase in applied frequency are due to space charge induced electric field distortion in the vicinity of the electrodes.

We have thus demonstrated the dc and ac (50–1000 Hz) breakdown characteristics of insulating materials, both in experiments and numerical simulations. Notably, the electrical breakdown of insulating materials is modulated by space charges. Generally, homo-charges are formed in a dc breakdown process, which reduce the electric strength in the vicinity of the sample-electrode interface and enhance electric strength in the middle of the bulk. In contrast, both positive and negative charges are accumulated within 0–2 μm in the vicinity of the electrodes under ac stress. These accumulated charges induce a significant electric field distortion that greatly enhances inner electric strength in the vicinity of the electrodes, which lead to the comparatively low breakdown strengths. This distortion is strengthened with applied frequency, which is responsible for the decreasing breakdown strength with applied frequency. In conclusion, dc breakdown initializes in the middle bulk of the material whilst ac breakdown initializes in the vicinity of the sample-electrode interface.

As the mechanisms of dc and ac breakdown are now revealed, it provides a new possible method in analysing and explaining breakdown phenomenon. Moreover, possible quantitative methods in modifying space charge distribution, like oxidation and fluoridation, should be further developed and emphasized as important approaches to control and improve the breakdown performance of insulating materials.

## Additional Information

**How to cite this article**: Li, S. *et al.* Space Charge Modulated Electrical Breakdown. *Sci. Rep.* **6**, 32588; doi: 10.1038/srep32588 (2016).

## Supplementary Material

Supplementary Information

## Figures and Tables

**Figure 1:**
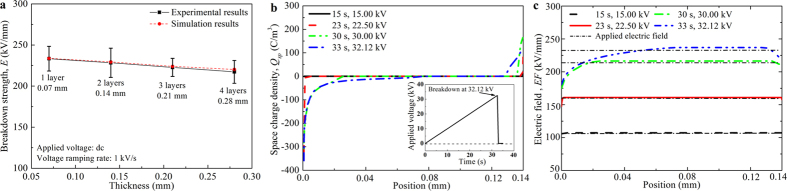
Space charge dynamics and breakdown strengths under dc stress. (**a**) Experimental and simulated breakdown strengths of 1–4 layer oil impregnated paper under dc stress, (**b**) space charge distribution as a function of position in a dc breakdown process of two-layer oil impregnated paper, (**c**) electric field distribution as a function of position in a dc breakdown process of two-layer oil impregnated paper.

**Figure 2:**
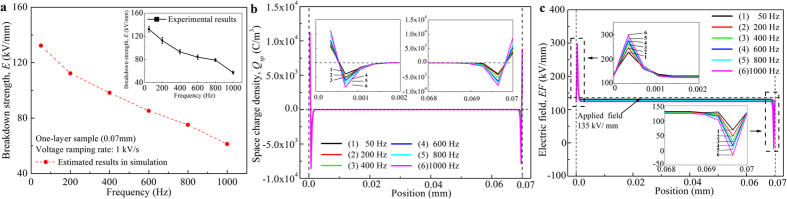
Space charge dynamics and breakdown strengths under ac stress. (**a**) Experimental and simulated breakdown strengths of one-layer oil impregnated paper under 50–1000 Hz ac stress, (**b**) space charge distribution as a function of position at applied electric strength of 135 kV/mm in 50–1000 Hz ac breakdown processes of one-layer oil impregnated paper, (**c**) electric field distribution as a function of position at applied electric strength of 135 kV/mm (at phase angle of 90°) in 50–1000 Hz ac breakdown processes of one-layer oil impregnated paper.
