# Supplementary material for: Space Charge Modulated Electrical Breakdown
Source: Sci Rep. 2016 Sep 7;6:32588. doi: 10.1038/srep32588 (PMC5013471; doi:10.1038/srep32588)
Supplement: Supplementary Information [file srep32588-s1.docx]

**Space Charge Modulated Electrical Breakdown**

Shengtao Li^1†^, Yuanwei Zhu^1‡^, Daomin Min^1^, George Chen^1,2^

^1^State Key Laboratory of Electrical Insulation and Power Equipment,

Xi'an Jiaotong University, Xi'an 710049, China

^2^School of Electronics and Computer Science

University of Southampton, Southampton SO17 1BJ, UK

*Email: [sli@mail.xjtu.edu.cn](mailto:sli@mail.xjtu.edu.cn), zhuyw890109@stu.xjtu.edu.cn

**Supplementary Information**

# **The electric conduction of oil impregnated paper at high fields**

The space charge dynamics of a typical insulating material (the oil impregnated paper) were numerically simulated in this paper. The selection of simulating model and the migration of space charges are closely related to the electric conduction of the material [1]. Therefore, the mechanism of electric conduction of oil impregnated paper was first investigated.

In our experiments, one-layer oil impregnated paper (0.07 mm) was fixed by cylindrical electrodes (*φ* = 25 mm). In order to exclude the influence of polarization current, the conduction currents were measured 6000 s after the application of dc voltage. The test results are shown in Fig. S1.


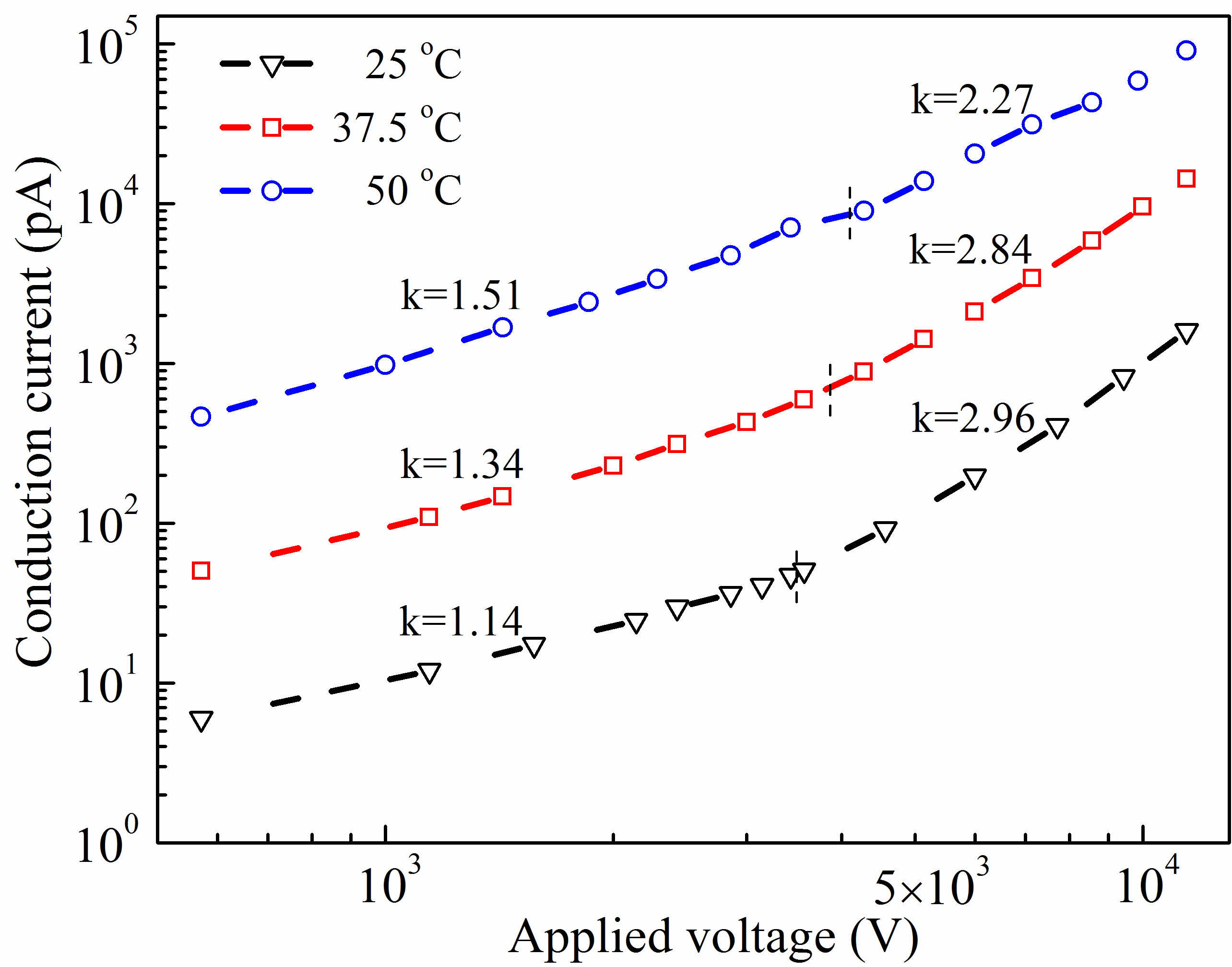


Figure S1. Electric conduction of one-layer oil impregnated paper at high fields (0.5-10 kV)

at temperatures of 25, 37.5, and 50 ^o^C

The existing mechanisms of electric conduction at high fields are the energy band model, hopping conduction model [2] and space charge limited current (SCLC) model [3]. The cellulose in insulating paper is composed of cross-connected crystalline and amorphous regions [4]. As a result, the distribution of cellulose atoms is regularly arranged in small localized crystalline regions; however, in larger regions, the atomic distribution becomes irregular. Therefore, the energy bands formed by regular arrangement of atoms can only exist in each small localized area. In larger amorphous regions, charge carriers cannot migrate freely as in the conduction band in crystalline regions. Consequently, it is generally acknowledged that the high-field conduction of oil impregnated paper does not conform to the energy band model.

For SCLC-based electric conduction, there is a strong criterion which demonstrates the correspondence of applied voltage (V) versus conduction current (J), as shown in Fig. S2. It can be observed that a transition voltage, *V_tr_*, which defines the space charge limited current, dominates over the Ohmic component in the experimental plot of log(J) versus log(V). Moreover, a voltage at which the slope changes from unity to two can be observed.


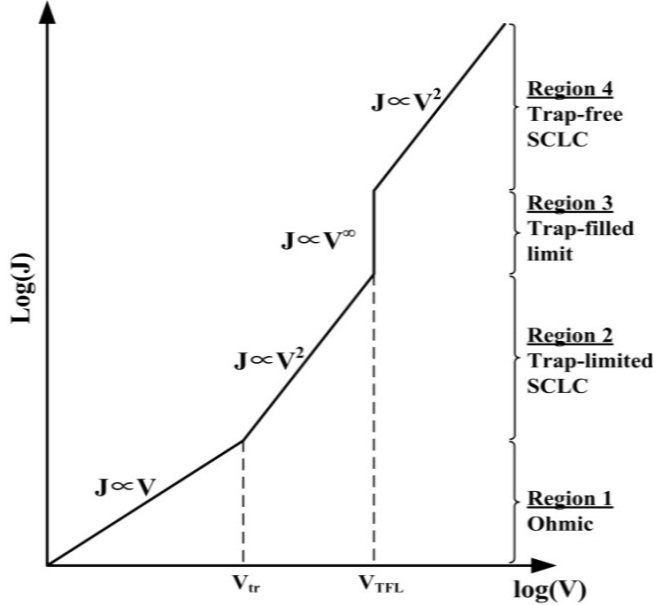


Figure S2. Schematic of space charge limited current-based electric conduction

From a joint analysis of Fig. S1 and Fig. S2, it can be concluded that the SCLC doesn’t dominate the high-field conduction of oil impregnated paper, as the miss-observation of the slope of two in Fig. S1.

Considering the high-field term, the current density in the hopping conduction model can be expressed as,

$J=\sigma_{0}\exp(-\frac{E_{T}}{k_{B}T})\sinh(\frac{d\left|EF\right|}{2k_{B}T})EF$ (1)

where *σ*_0_ is the conduction coefficient in S/m; *E_T_* is the conduction barrier height (charge hopping barrier height) in eV; *k_B_* is the Boltzmann constant; *T* is temperature in K; *d* is the charge hopping distance in m; and *EF* is the applied electric field in V/m.

We re-plot the results of high field conduction at 25 ^o^C on a time-domain scale, and employ Equation (1) in fitting the curve, as shown in Fig. S3.


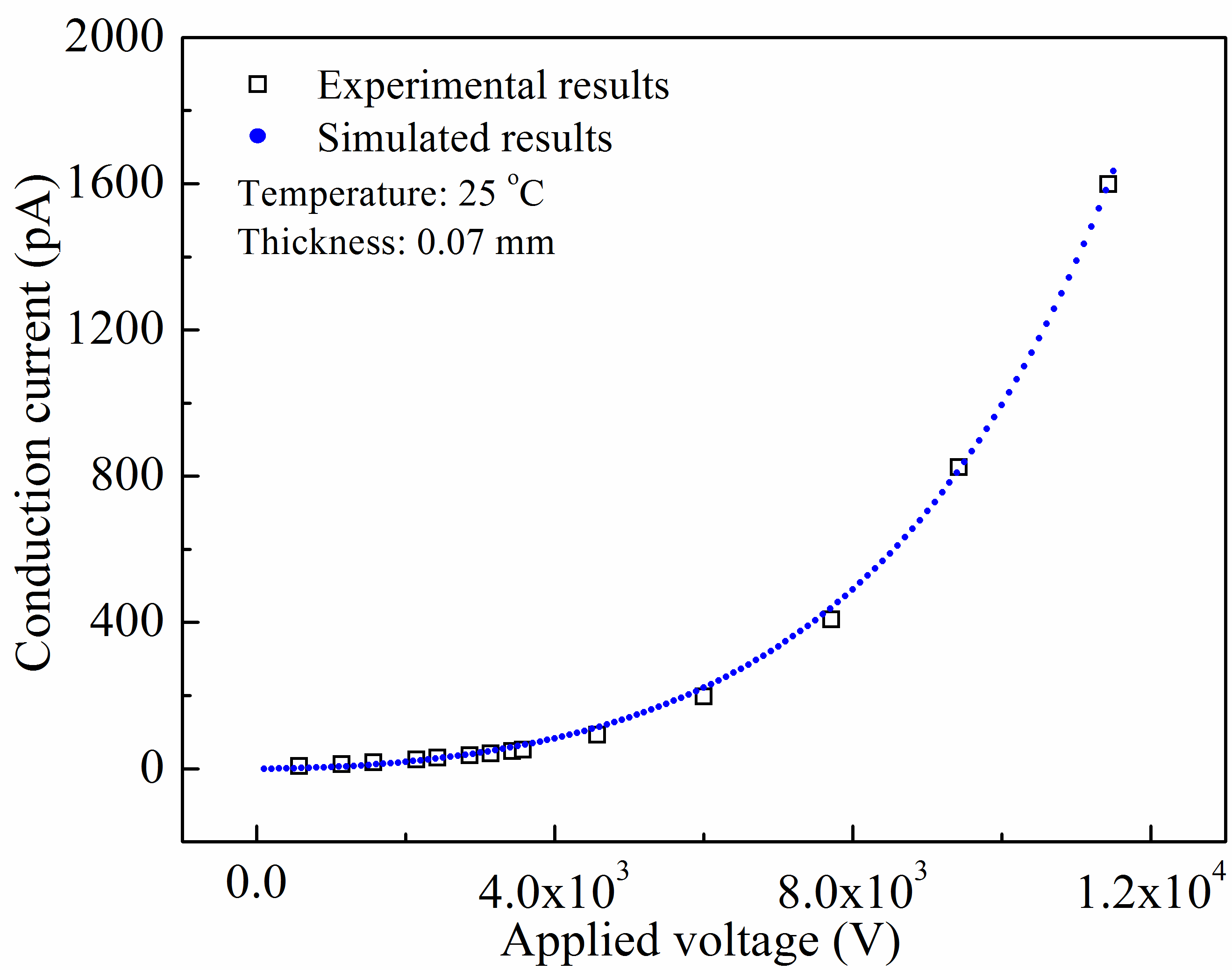


Figure S3. Experimental and simulated curves of high field electric conduction

of oil impregnated paper at 25 ^o^C

It is observed that the simulated curve is consistent with the experimental results. Therefore, based on the theoretical analysis and the experimental results, we conclude that high-field conduction of oil impregnated paper obeys the law of hopping conduction. The hopping conduction model is employed in our numerical simulations.

# **The model and selection of assigned parameters in numerical simulations**

When a voltage is applied to an insulating material, charges can be injected from the electrodes. We utilize a BCT model to investigate space charge dynamics in oil impregnated paper, as shown in Fig. S4. We consider a system consisting of an oil impregnated paper sample with the thickness of *L* clamped by two electrodes. A one-dimensional coordinate, x, is set up for the sample, where *x*=0 corresponds to the interface between the material and the left electrode, while *x*=*L* corresponds to the interface between the material and the right electrode.


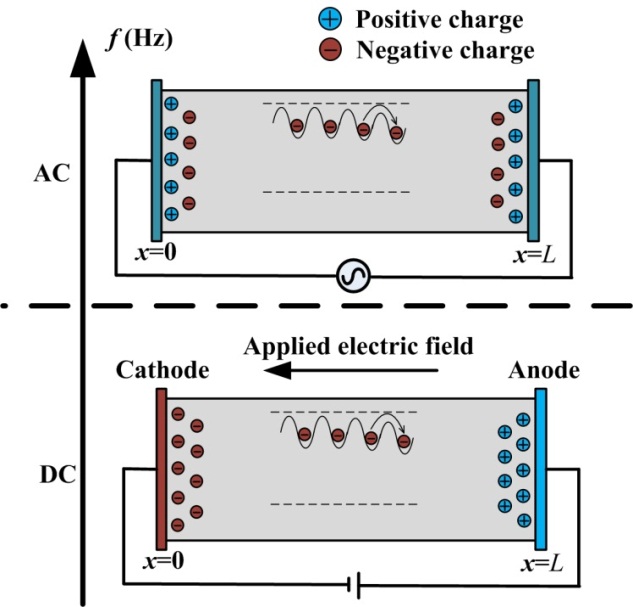


Figure S4. Schematic of a model of bipolar charge injection and transport in oil impregnated paper

under dc and ac voltages

According to the discussion above, the electric conduction of oil impregnated paper at high fields (＞10^7^ V/m) obeys hopping conduction. At room temperature, the charge injection follows Schottky thermionic emission, as,

 (1)

where *j*_in_ is Schottky emission current in A/m^2^; *A* is the Richardson constant; *T* is the absolute temperature in K; *ϕ*_in_ is the Schottky injection barrier in eV; *EF* is the electric field in V/m; *k_B_* is the Boltzmann constant; *q_e_* is the elementary charge in C; *ε*_0_ is the vacuum permittivity in F/m; and *ε*_r_ is the dielectric constant (*ε*_r_ = 2.0 for oil impregnated paper).

The charges in insulating materials are governed by a set of self-consistent equations.

1. Charge transport equation,

$j_{c}\left(x,t\right)=Q\left(x,t\right)\mu EF\left(x,t\right)$ (2)

1. Charge advection-reaction equation,

$\frac{\partial Q\left(x,t\right)}{\partial t}+\frac{\partial j_{c}\left(x,t\right)}{\partial x}=S(x,t)$ (3)

1. Poisson’s equation,

 (4)

1. Charge dynamic equation,

$S(x,t)=RQ_{e}(x,t)Q_{h}(x,t)$ (5)

where *j*_c_ is the conduction current in A/m^2^; *x* is the position of sample in m; *t* is the time in s; *Q* is the charge density in C/m^3^; *μ* is the charge mobility in m^2^/Vs; *φ* is the potential of the charged material in V; and *S* is the source term.

For the thickness-dependent dc breakdown, the interfacial polarization between oil impregnated paper layers was ignored in the simulations, as the quantity of accumulated charges at the interface is extremely small when compared to that in the vicinity of the electrodes [5].

In numerical simulations, the parameters (in Equations. (1) - (5), closely related to the material) were obtained through experiments of high field conduction and isothermal surface potential decay (ISPD). The experimental processes of ISPD and the data processing are shown as below.

In order to obtain the conduction barrier height for both positive and negative charges, ISPD experiments of oil impregnated paper samples are conducted at 20^o^C (the temperature of conducting the breakdown tests). The schematic of the corona charging system used in ISPD experiments is shown in Fig. S5.


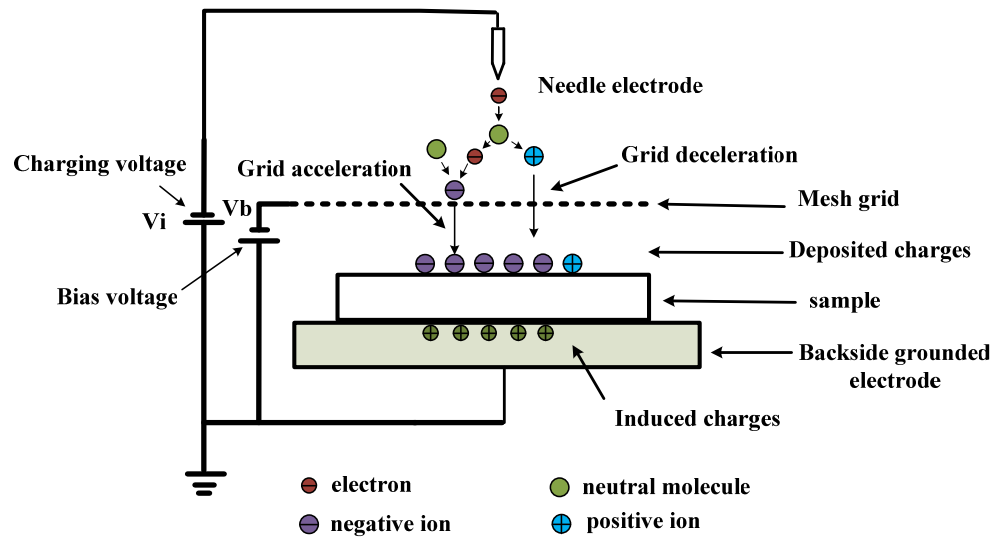


Figure S5. The schematic of the corona charging system [6]

In these experiments, the charging voltage was 10 kV, the bias voltage was 4 kV, the charging duration was 300 s and the discharging duration was 5000 s. The results are shown in Fig. S6.





Figure S6. ISPD results of oil impregnated paper with positive and negative charging at 20 ^o^C

It is observed that there occurs a quicker decay process for negative charges, which indicates a lower conduction barrier height and greater charge mobility. By employing the plot of d*V*/d*t* versus time, the transit time *t_T_* of the ISPD curves were obtained [7], and the results are shown in Table S1.

Table S1. Transit time of negative and positive charges in oil impregnated paper

at temperatures of 20 ^o^C

| **Temperature** | **Transit time (s)** | |
| --- | --- | --- |
|  | **Positive charge** | **Negative charge** |
| 20 ^o^C | 41.79 | 30.87 |

The charge mobility can be expressed by Equation (6),

$\mu=\frac{L^{2}}{t_{T}V_{0}}$ (6)

where *L* is the sample thickness in m; *t*_T_ is the transit time in s; *V*_0_ is the initial surface potential of the material in V.

According to Equation (6), the mobility of positive and negative charges can be obtained, and it is shown in Table S2.

Table S2. The mobility of positive and negative charges in oil impregnated paper

at temperatures of 20 ^o^C

| **Temperature** | **Mobility (m^2^V^-1^s^-1^)** | |
| --- | --- | --- |
|  | **Positive charge** | **Negative charge** |
| 20 ^o^C | 3.77×10^-14^ | 5.14×10^-14^ |

The conduction barrier height was obtained based on an improved Simmons’ model [8], the details of which were documented and demonstrated in our previous investigation [6]. In calculating the conduction barrier height, the ISPD curves at 20 ^o^C (the temperature at which the breakdown tests were conducted) were carefully fitted, as shown in Fig. S6. In the curve fitting, a double exponential decay equation was applied, as,

 (7)

The fitting coefficients R^2^, are 0.98983 and 0.99854 for positive and negative charging sequentially, which indicates that the ISPD curves were accurately fitted by employing the double exponential decay equation. The fitting parameters are shown in the inserted table in Fig. S6.

The conduction barrier height and charge density can be expressed as [6],

$E_{T}=k_{B}T\ln(\nu_{ATE}t)$ (8)

 (9)

where *E_T_* is conduction barrier height in eV; *ν*_ATE_ is the attempt-to-escape frequency, which can be expressed as, *ν*_ATE_=(*k_B_T*)^3^/*dh*^3^*ν* ^2^, here *h* is the Planck constant, the value of *d* is 6, and *ν* is the vibration frequency around the defects at the orthogonal plane flaw in the direction of motion.

Based on Equation (8) and Equation. (9), the distributions of conduction barrier heights for positive and negative charges were obtained. The results are shown in Fig. S7.





Figure S7. Distributions of conduction barrier height in oil impregnated paper at 20 ^o^C

It is observed that the conduction barrier height in oil impregnated paper is around 0.68-0.90 eV. Compared to negative charges, the conduction barrier height for positive charges is larger, at a peak value of 0.78 eV. The peak barrier height for negative charges is 0.76 eV.

The measured conduction barrier height varies with the testing methods. In this paper, the conduction barrier height was measured in the range of 0.68-0.90 eV by ISPD experiment. Tang et. al. measured the conduction barrier height (mentioned as trap energy in the paper) of the same type of oil impregnated paper by PEA method, and concluded that the conduction barrier is in the range of 0.37-0.53 eV [9]. Wei et. al. measured the conduction barrier height by thermally simulated current (TSC) method, and obtained the result of 0.60-0.90 eV [10]. Base on the experimental results of ISPD and high-field conduction, and in combination with the results obtained in literature described above, the assigned parameters in the numerical simulation were obtained, as shown in Table S3.

Table S3. The assigned values of the parameters used in the bipolar charge transport simulations

| Parameter | Unit | Value |
| --- | --- | --- |
| Ac frequency | Hz | 50-1000 |
| Voltage ramping rate | V/s | 1000 |
| Electron hopping distance | m | 2.56×10^-9^ |
| Hole hopping distance | m | 1.38×10^-9^ |
| Electron hopping barrier height | eV | 0.68 |
| Hole hopping barrier height | eV | 0.78 |
| Temperature | K | 300 |
| Sample thickness | m | 7×10^-5^ |
| Spatially discrete grid |  | 200 |
| Calculating time step | s | 1×10^-6^ |
| Intrinsic breakdown strength | V/m | 2.33×10^8^ |

# **The sample preparation**

The insulating paper used in the experiments is provided by Xi'an Power Transformer Co., China. The thickness of the insulating paper is 70 μm. The transformer oil used is Karamay 25^#^ transformer oil, which has been widely used in various types of transformers in China.

The procedure of preparing the oil impregnated paper samples was as follows. The insulating paper was first cut into 60 mm × 60 mm square pieces and placed into a 100 Pa, 80 ^o^C oven for 48 h to remove moisture. After that, the insulating paper was kept at 25 ^o^C and 50 Pa for 24 h. After this treatment, the water content in insulating paper was ≤ 0.2%, which satisfied the engineering requirements. The Karamay 25^#^ transformer oil was first filtered with a double-stage vacuum oil purifier. Then, the filtered oil was heated at 80 ^o^C and 50 Pa for 4 h. After the treatment, the water content in transformer oil was ≤ 5 ppm, the air content was ≤ 0.1 %, and the impurity granularity was ≤ 3μm.

After considering a similar process with comparable practical engineering procedures, the vacuum oiling process was as follows. The insulating paper was firstly put into a clean oiling chamber, and dried again in vacuum (50 Pa) at 115 ^o^C for 4 h. After that, the Karamay 25^#^ transformer oil was injected. Finally, the vacuum oiling chamber with OIP samples inside was kept at 90 °C for 8 h.

# **The breakdown test and simulation**

The breakdown test system can provide up to 100 kV dc, 100 kV 50 - 1000 Hz ac and 100 kV 1.2/50 μs negative lightning impulse voltages.

In breakdown tests, a brass spherical electrode was employed, which could insure a uniform electric field distribution and reduce physical injury to the surface of oil impregnated paper samples. The geometry of the electrode is shown in Fig. S8. In breakdown tests, the spherical electrode with oil impregnated paper sample was immersed into fresh Karamay 25^#^ transformer oil. The upper sphere was connected to the high voltage source; the lower electrode was grounded.


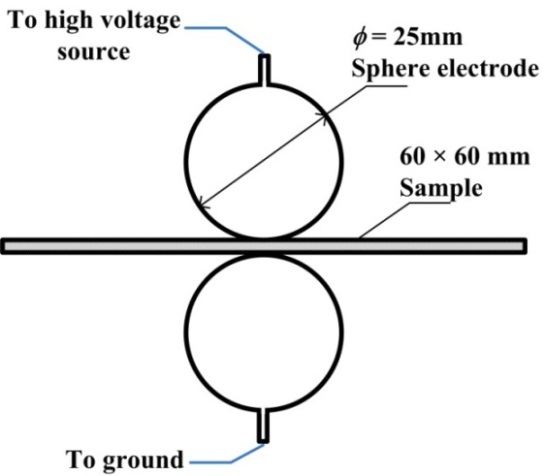

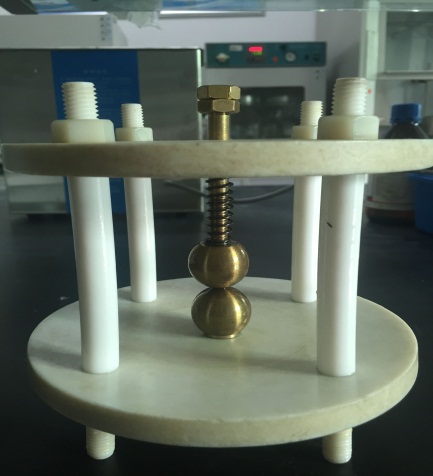


(a) schematic (b) picture

Figure S8. The sphere-sphere electrode employed in breakdown tests

During the breakdown tests, a dc (or ac) voltage was applied to the oil impregnated paper sample and elevated at a ramping rate of 1 kV/s until breakdown. The waveform of applied dc (or ac) voltage is shown in Fig. S9. When breakdown occurred, the breakdown voltage was recorded. In order to ensure the reliability of experimental data, each group of test was performed for 15 times. The ac voltage was recorded in peak-to-peak value.


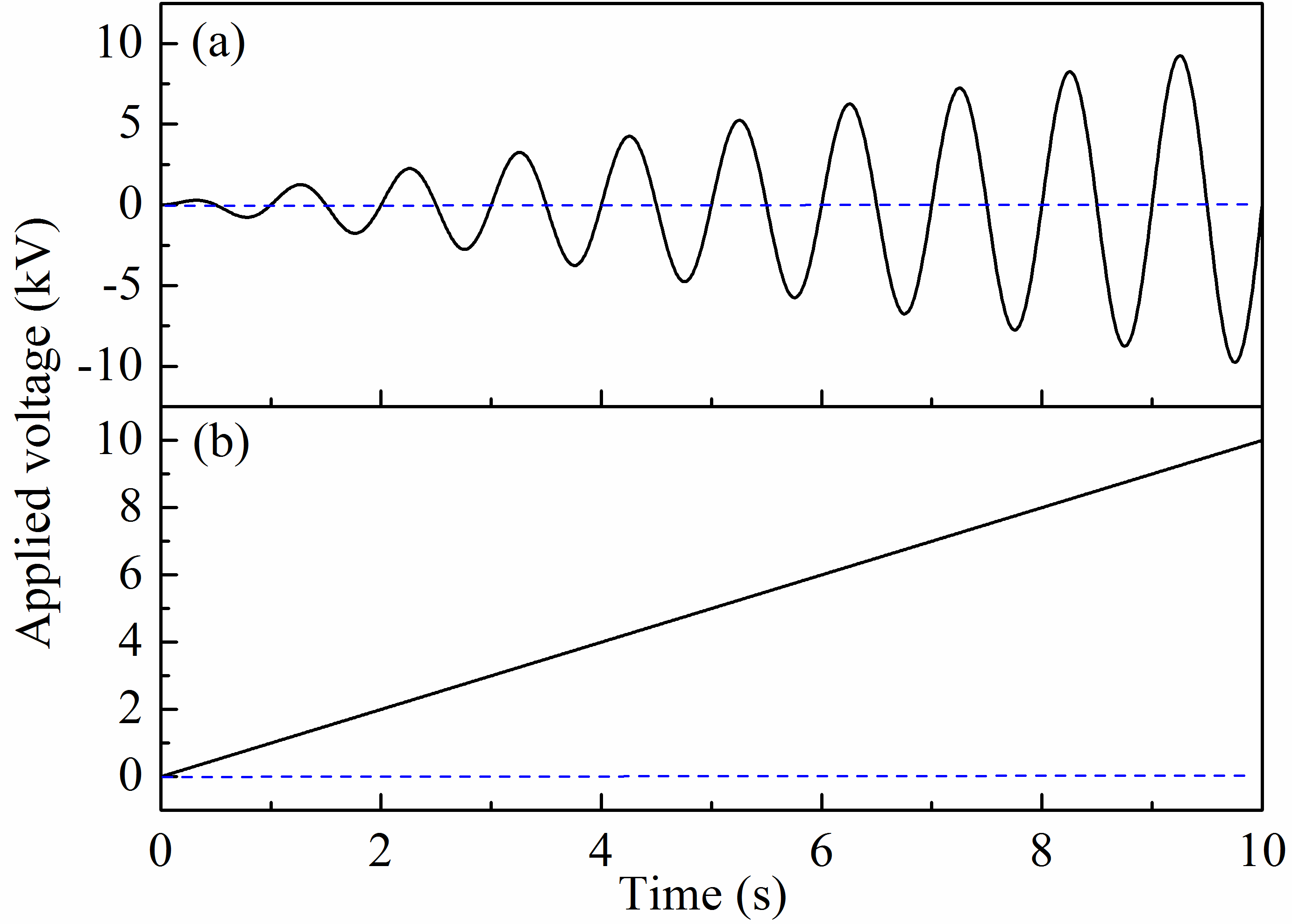


Figure S9. Schematic of the ramping voltage (a) ac voltage with ramping rate of 1 kV/s

(b) dc voltage with ramping rate of 1 kV/s

During the simulation, the dc breakdown was recorded when the maximum electric field in the bulk of the sample reaches the pre-set intrinsic breakdown strength of the material. In order to more clearly demonstrate the space charge accumulations with the variation of frequency under ac stress, it was assumed that no breakdown occurs before the applied voltage reaches the 50 Hz ac breakdown voltage. As it was concluded in the paper that the electric field distortion introduced by the space charges determines the ac breakdown of insulting materials, the ac breakdown strength with the variations of applied frequency are estimated by the difference in maximum electric field distortion between 50 Hz and the now-applied frequency.

# **Supplementary Reference**

1. Min, D., Cho, M. Khan, A. R. & Li, S. Charge transport properties of dielectrics revealed by isothermal surface potential decay, *IEEE Trans. Dielectr. Electr. Insul.* **19**, 1465-1473 (2012).
2. Das-Gupta, D. K. Conduction mechanisms and high-field effects in synthetic insulating polymers, *IEEE Trans. Dielectr. Electr. Insul.* **4**, 149-156 (1997).
3. Grinberg, A. A., Luryi, S., Pinto, M. R. & Schryer, N. L. Space-charge-limited current in a film, *IEEE Trans. Elec. Devi.* **36**, 1162-1170 (1989).
4. Mazeau, K. & Heux, L. Molecular dynamics simulations of bulk native crystalline and amorphous structures of cellulose. *J. Phys. Chem. B* **107,** 2394-2403 (2003).
5. Huang, M., Zhou, Y., Chen., W., Lu, L., Jin, F. & Huang, J. Space charge dynamics at the physical interface in oil-paper insulation under dc voltage, *IEEE Trans. Dielectr. Electr. Insul.* **22**, 1739-1746 (2015).
6. Li, J., Zhou, F., Min, D., Li, S. & Xia, R. The energy distribution of trapped charges in polymers based on isothermal surface potential decay model, *IEEE Trans. Dielectr. Electr. Insul.* **22**, 1723-1732 (2015).
7. Ziari, Z, Sahli, S. & Bellel, A. Mobility dependence on electric field in low density polyethylene (LDPE), *M. J. Condensed Matter* **12** 223-226 (2010).
8. Simmons, J. G. & Tam, M. C. Theory of isothermal currents and the direct determination of trap parameters in semiconductors and insulators containing arbitrary trap distributions, *Phys. Rev. B.* 7 3706-3713 (1973).
9. Tang, C., Liao, R., Chen, G. & Yang, L. Research on the feature extraction of DC space charge behavior of oil-paper insulation, *Sci. China Technol. Sc.* **54** 1315-1324 (2011).
10. Wei, Y., Zhu, M., Li, Y., Zhao, L., Deng, J., Mu, H. & Zhang, G. Partial discharge characteristics and trap parameters of aged oil-impregnated paper, *IEEE Trans. Dielectr. Electr. Insul.* **22**, 3442-3450 (2015).
